# Pigmented basal cell carcinoma of the anus: a rare entity with diagnostic challenges

**DOI:** 10.1093/jscr/rjae554

**Published:** 2024-08-28

**Authors:** Kevin Joseph Fuentes-Calvo, Cielo Scarlet Silva-Ramos, Sara Fernanda Arechavala-López, Fernando Aguilar-Ruiz, Luis Felipe Arias-Ruiz, Mario Trejo-Ávila

**Affiliations:** General Surgery Department, Hospital Médica Sur, Mexico City, Mexico; General Surgery Department, Hospital Médica Sur, Mexico City, Mexico; Hospital Médica Sur, México City, Mexico; General Surgery Department, Hospital Médica Sur, Mexico City, Mexico; Department of Anatomic Pathology, Fundación Clínica Médica Sur, Mexico City, Mexico; Department of Colon and Rectal Surgery, Hospital Médica Sur, Mexico City, Mexico

**Keywords:** basal cell carcinoma, basaloid, squamous cell carcinoma, anus, histologic type

## Abstract

Anal cancer is uncommon, comprising 2.2% of gastrointestinal cancers. Squamous cell carcinoma (SCC) is the most common; while perianal basal cell carcinoma (BCC) is rare, representing only 0.2% of anorectal malignancies. BCC, associated with sun exposure and immunosuppression, often resembles benign conditions and manifests as perianal ulcers or masses. Histologically, BCC exhibits basaloid tumor cells with distinct patterns. Despite its rarity, accurate diagnosis is crucial. We expose a case study of a 59-year-old male, previously healthy, that presented with hematochezia and perianal pain, leading to a diagnosis of lower gastrointestinal bleeding. Colonoscopy was needed, and a biopsy revealed an ulcerated, indurated lesion involving the left lateral hemorrhoidal bundle, diagnosed as pigmented basaloid carcinoma. Microscopic examination showed malignant nests of cells with peripheral nuclear palisading, melanocytes, and melanin pigment. Immunohistochemistry confirmed positivity for p63, CK5/6, and BCL2. Respect the treatment, due to the involvement of the anal sphincteric muscle, radiotherapy was chosen.

## Introduction

Anal cancer, which affects the anus, anal canal, or anorectum, represents ~2.2% of all gastrointestinal malignancies. Squamous cell carcinoma (SCC) is the most common type of anal cancer, while perianal basal cell carcinoma (BCC) is the rarest among all anorectal malignancies [1]. BCC, comprises ~0.2% of anorectal neoplasms. Traditionally, they have been divided into well-differentiated keratinizing and non-keratinizing (cloacogenic, basaloid, and transitional) tumors. These tumors primarily originate in the junctional zone between the endoderm and ectoderm, known as the cloacogenic zone, located in the upper portion of the anal canal. This location is attributed to the inherent epithelial instability of this zone. The term ‘basaloid’ is used because these tumors resemble BCC. Coined by Wittoesch *et al.* in 1957, the term ‘basaloid’ delineates tumors of the anal canal exhibiting histological parallels to BCC of the skin.

These carcinomas are characterized by marked histological undifferentiation and a heightened proliferation rate [2]. BCC, is the most common cancer in the United States and it is associated with immunosuppression and sun exposure [3]. BCCs comprise ~0.2% of anorectal neoplasms and can mimic anal cysts and hemorrhoids. Macroscopically, appear as a perianal ulcer or a mass with raised edges and ulceration. BCCs feature cells with hyperchromatic nuclei and scant to moderate eosinophilic cytoplasm (basaloid tumor cells). Tumor cells group in nested or nodular patterns, which show the characteristic peripheral nuclear palisading. The histological features of BCC and SCC often overlap [4]. However, certain features may aid in the differential diagnosis and immunohistochemistry markers that may aid a pathologist to distinguish between BCC and SCC [2, 3, 5]. Owing to its infrequency, we present a case of a 59-year-old, previously healthy male with hematochezia, subsequently diagnosed with anal basaloid carcinoma involving the hemorrhoidal bundles, alongside a comprehensive literature review.

## Case report

This is about a 59-year-old man with no relevant medical history, except for a 10-year history of tobacco use, smoking two cigarettes per day. He began experiencing hematochezia and perianal pain during defecation, prompting him to seek emergency care. Previous doctors recommended treatment for hemorrhoidal disease. After several attempts of treatment, patient decided to seek specialty care. Routine laboratory tests were performed, showing no abnormalities. The colon and rectal surgery department evaluated him and diagnosed him with lower gastrointestinal bleeding. As part of the diagnostic evaluation, a colonoscopy with biopsy was performed, revealing an ulcerated, indurated, and friable lesion in the perianal region, at the 9 o’clock position, involving the left lateral hemorrhoidal bundle.

**Figure 1:**
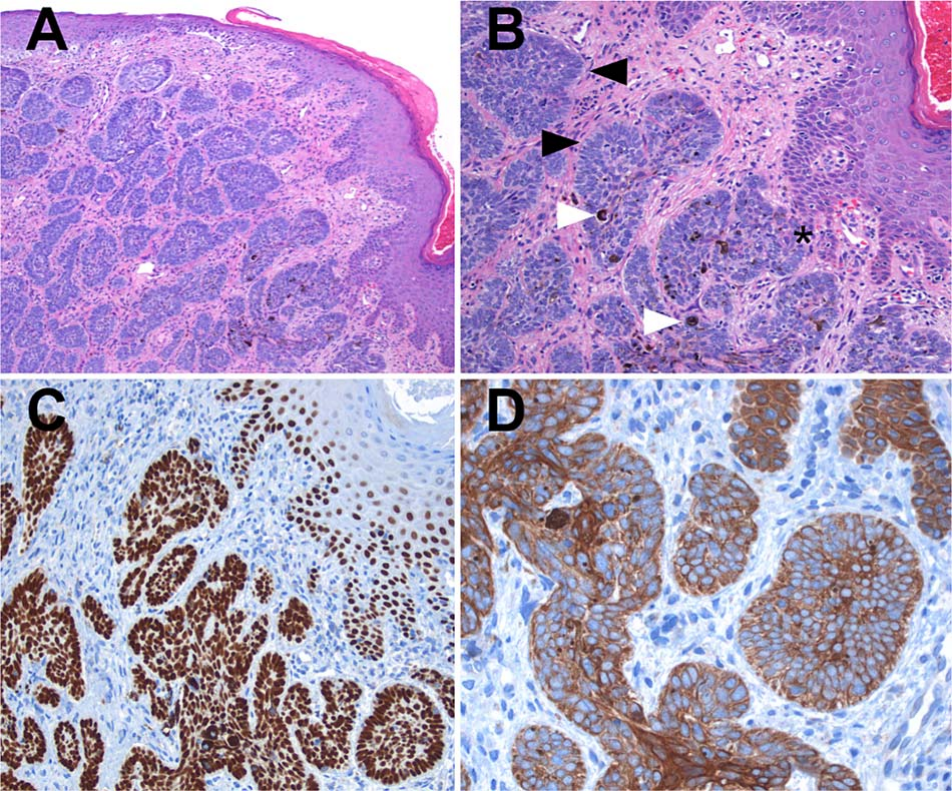
Hematoxylin and eosin (H&E) staining. (A) Display of normal stratified flat epithelium of the skin with a subjacent neoplasm featuring small nests. (B) Higher magnification reveals the tumor arising from the basal layer of the skin (*). The nests exhibit characteristic peripheral nuclear palisading (black arrows), and both melanin pigment and melanocytes are visible (white arrows). (C) Both the tumor and adjacent skin cells exhibit strong nuclear positivity for p63. (D) Tumor cells demonstrate strong positivity for high molecular weight cytokeratins (CK5/6).

**Figure 2:**
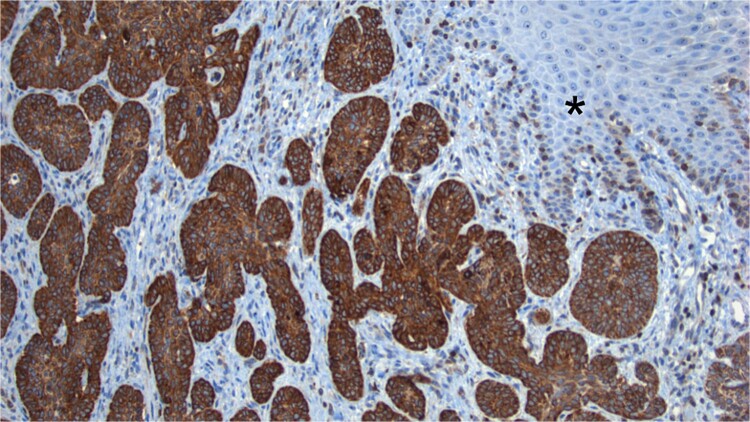
BCL2 Staining. Tumor cells show intense positivity for BCL2, contrasting with the negativity observed in the normal skin (*).

The sample was sent to pathology for consultation, where the provisional diagnosis was identified as ‘pigmented basaloid carcinoma.’ Microscopically, the hematoxylin and eosin-stained sections revealed a malignant tumor characterized by nests of cells with scant to moderate cytoplasm and basophilic nuclei, exhibiting peripheral nuclear palisading. Melanocytes and melanin pigment were observed within the tumor nests and stroma. Immunohistochemical tests showed positive results for p63 and CK5/6 (Fig. 1), and negative results for HMB-45. Additionally, intense and diffuse positivity for BCL2 was noted (Fig. 2). The final diagnosis confirmed a pigmented basal cell carcinoma with infiltrative, micronodular, and ulcerative patterns.

**Figure 3:**
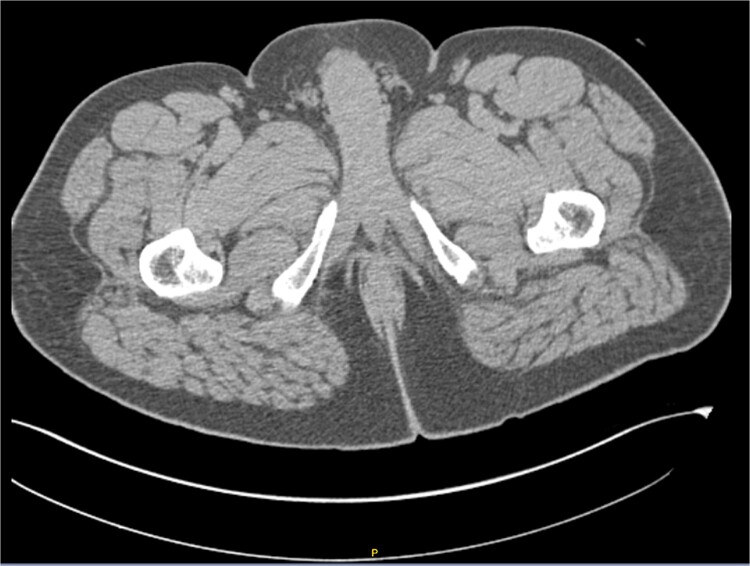
Computed tomography (CT) scan with intravenous contrast (IV). Hepatic cystic lesions, with diameters of up to 5 mm, which did not enhance with contrast. Additionally, it was observed that the perianal region showed no fat stranding or increased volume, and no lymphadenopathy was detected.

After receiving the pathology report, an abdominal computed tomography (CT) scan with intravenous contrast was ordered (Fig. 3). The analysis revealed diffuse distribution of hepatic cystic lesions, with diameters of up to 5 mm, which did not enhance with contrast. Additionally, it was observed that the perianal region showed no fat stranding or increased volume, and no lymphadenopathy was detected.

**Figure 4:**
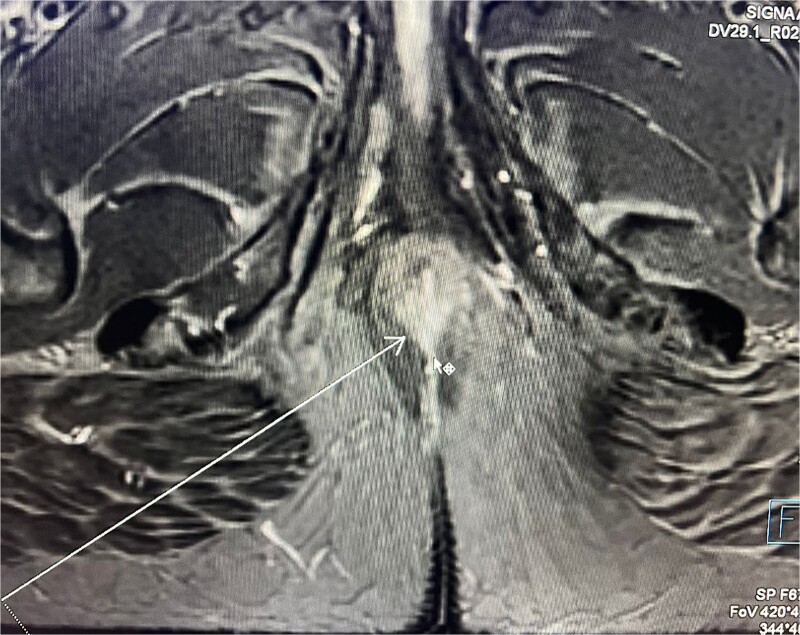
Abdominal and pelvic magnetic resonance imaging. The white arrow points to a 16 x 22 mm mass in the perianal region, where asymmetric thickening affecting both the external and internal sphincters is evident, located just at the anodermic junction and extending into the levator ani muscles.

Consequently, an abdominal and pelvic magnetic resonance imaging (MRI) was performed (Fig. 4), which revealed a 16 x 22 mm mass in the perianal region, characterized by asymmetric thickening affecting both the external and internal sphincters, just at the anodermic junction, with extension into the elevator ani muscles.

**Figure 5:**
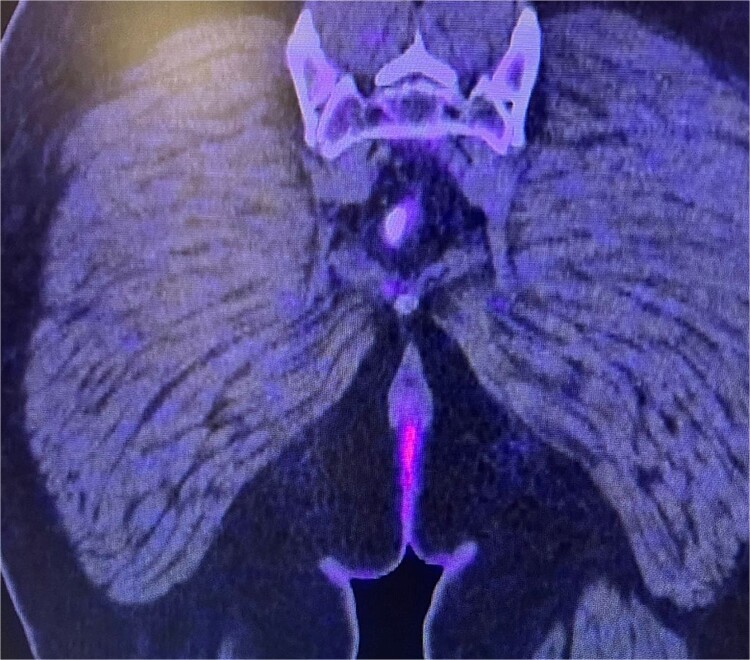
Abdominal and pelvic PET-CT, coronal section. Image showing perianal region without uptake and absence of adenopathy.

Subsequently, a consultation with medical oncology was requested, which recommended performing a positron emission tomography (PET-CT) scan (Fig. 5) to assess the presence of metastatic disease. However, the report only indicated the presence of hypodense nodular images measuring 5 mm in segment VII of the liver, suggestive of cysts, as well as the absence of uptake in the perianal region and the lack of lymphadenopathy.

Subsequently, it was decided to discharge him from the hospital for further outpatient follow-up and to plan oncologic treatment.

Treatment was decided in a multidisciplinary team meeting (colorectal surgeon, oncologist, radiologist, and radiation oncologist). Due to the involvement of the anal sphincteric muscle and the extension into the elevator ani muscle, radiotherapy was chosen. Patient underwent radiation therapy with a dose of 50.4 Gy. Due to lack of obstruction, pre-radiation colostomy was not required.

**Figure 6:**
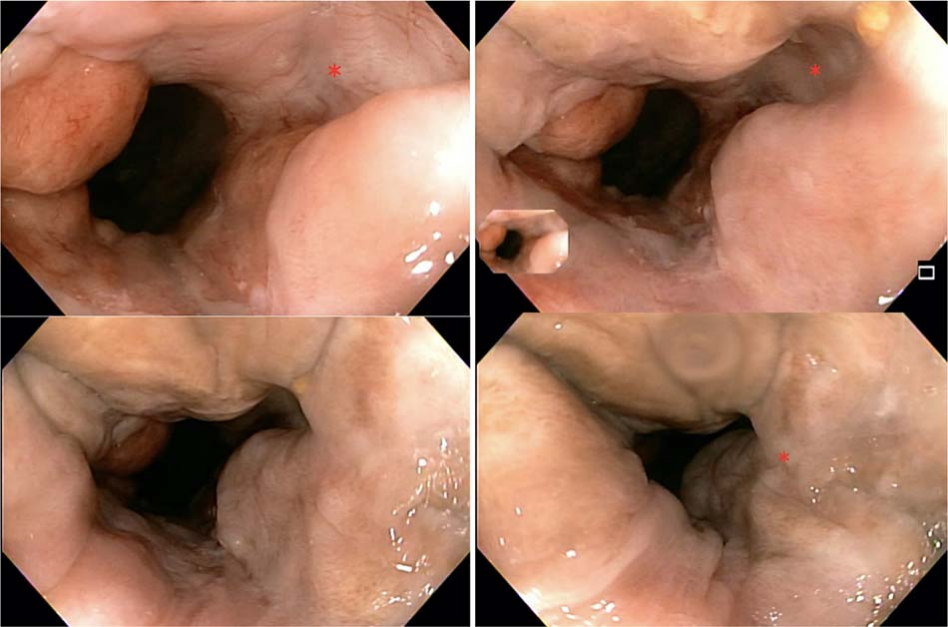
Colonoscopy findings. Image showing a flat white scar, telangiectasia, and absence of both ulcer and nodularity.

The patient was regularly follow-up a 4-month intervals, and no recurrence was found during a follow-up period of 12 months. Patient remained with good anorectal and sexual function. A 1 year follow-up colonoscopy was performed, finding complete response, with a flat white scar, telangiectasia and absence of both ulcer and nodularity (Fig. 6).

## Discussion

Anal cancer, which affects the anus, anal canal, or anorectum, represents ~2.2% of all gastrointestinal malignancies [3]. This disease tends to manifest in the sixth decade of life. Although relatively uncommon, its incidence is increasing due to various risk factors such as anal-genital infection with the human papillomavirus, immunosuppression related to human immunodeficiency virus or transplantation, and smoking habit [5–7], also, the advanced age and the male gender are considered independent risk factors. These tumors are classified into two categories based on their location: anal canal tumors or anal margin tumors.

Traditionally, they have been divided into well-differentiated keratinizing and non-keratinizing (cloacogenic, basaloid, and transitional) tumors. Within the non-keratinizing category lies the basaloid variant of SCC and BCC. These tumors primarily originate in the junctional zone between the endoderm and ectoderm, known as the cloacogenic zone, located in the upper portion of the anal canal. This location is attributed to the inherent epithelial instability of this zone. The term ‘basaloid’ is used because these tumors resemble BCC histologically [8].

BCC is the most common malignancy [9], yet it only comprises ~0.2% of anorectal neoplasms. Macroscopically BCC appear as a perianal ulcer or a mass with raised edges and ulceration. They can also mimic anal cysts and hemorrhoids [4].

All types of BCC feature cells with hyperchromatic nuclei and scant to moderate eosinophilic cytoplasm (basaloid tumor cells). Tumor cells group in nested or nodular patterns, which show the characteristic peripheral nuclear palisading [4].

The histological features of BCC and basaloid SCC often overlap, displaying the characteristics previously mentioned. This overlap can make it challenging for pathologists to distinguish between the two. However, certain features may aid in the differential diagnosis: (i) BCCs do not have a known precursor, whereas SCCs originate from anal squamous intraepithelial neoplasia, (ii) BCCs typically originate at the anal margin, while SCCs arise within the anal canal or at the anal margin, including up to 5 cm of the perianal skin, and (iii) BCC may be colonized by benign melanocytes –with melanin [10].

Immunohistochemistry can be beneficial when it comes to making the differential diagnosis, as BCC show positivity for Ber-EP4, BCL2 (67%–100%), SMA (78%–95%), CD117 (93%) and some neuroendocrine, such as CD56 (77%) and chromogranin A (27%–72%), while basaloid SCC is often negative for these markers [5, 7, 8, 11–13]. p16 and SOX2 are also useful, as they are often positive in SCC, while negative in BCC [11], p63 and CK5/6 are positive in nearly all cases of BCC and basaloid SCC, therefore useless to make the differential diagnosis.

Our case presented the typical morphology of a basaloid carcinoma, with differential diagnoses including BCC and basaloid SCC. Additionally, melanoma was ruled out due to a negative result for HMB-45 [7]. Given the remaining possibilities of BCC and SCC and considering the presence of melanin pigment and scattered melanocytes, the most likely diagnosis was BCC. SCCs are very rarely pigmented (~0.01% of all cases of SCC), whereas melanin and melanocytes are more commonly found in BCCs (6.5%–8.7%). Lastly, intense, and diffuse positivity for BCL2 strongly supports the diagnosis of BCC [11].

For the diagnosis, a thorough dermatologic examination at the time of diagnosis of perianal BCC should be performed to locate synchronous lesions, where there is a condition called nevoid basal cell carcinoma syndrome whereby patients develop multiple BCCs and potentially jaw, ovarian, and cardiac tumors [7]. So, the clinical presentation commonly includes rectal bleeding (45%) and anal pain or urgency (30%), while between 10% and 20% of patients present with extra-pelvic disseminated disease at the time of diagnosis. For patients who are eligible for surgery, the recommended initial treatment for BCC is a standard excision with a negative margin. In cases of high-risk primary BCCs, a surgical margin of 4–5 mm is suggested. Liu *et al.* recommend that the margin of resection range from 0.5 to 1.5 cm, which is greater than the 0.4 cm margin recommended by the National Comprehensive Cancer Network [7]. For recurrent lesions, a surgical margin of 6 mm or Mohs microscopic surgery is recommended. Also, hedgehog pathway inhibitors, such as vismodegib or sonidegib, can be utilized for patients with a nodal or distant recurrence.

For patients who are not suitable for surgery, definitive radiotherapy is the preferred treatment option. The recurrence rate was found to be 7.4%. Adjuvant radiotherapy has not been shown to be effective in the treatment of perianal BCC. Its use is controversial and should only be considered in the case of extensive disease.

The five-year survival rate in stage IV is 20.9% for squamous cell carcinomas and 7.4% for non-squamous cell carcinomas [12]. The standard treatment for metastatic anal carcinoma involves cisplatin-based chemotherapy in combination with 5FU and paclitaxel, along with radiotherapy for local treatment [8]. Detection of overexpression of the epidermal growth factor receptor (EGFR) in anal carcinoma suggests a potential benefit from anti-EGFR therapies such as cetuximab [12].

## Conclusion

Identifying a BCC tumor represents a challenge due to its resemblance to other anal diseases and because it is a rare disease with limited cases presented worldwide. However, for the diagnosis, we need confirmation by pathology, where it is confirmed by staining with BCL2. It should be remembered that the standard treatment for most patients is the local excision with clear margins. On the other hand, for patients who are not surgical candidates, we have treatment alternatives such as radiotherapy or hedgehog pathway inhibitors. In our case, due to the involvement of the anal sphincteric muscle and the extension into the elevator ani muscle, radiotherapy was chosen. Due to lack of obstruction, pre-radiation colostomy was not required. In the follow-up, the patient conserved a good quality of life, and in the last colonoscopy was performed, finding complete response, with a flat white scar, telangiectasia and absence of both ulcer and nodularity. This correlates with the treatment proposals described in the international literature.

As anal BCC is a rare disease with limited information and because of the presentation along with the pathology report with immunohistochemistry and staining makes this case unique, where the field of opportunity is to understand the behavior of the disease, its clinical course and its treatment since there are several therapies that are under investigation.
